# Phase II trial of sodium phenylbutyrate and taurursodiol in Wolfram syndrome

**DOI:** 10.1172/JCI198519

**Published:** 2026-05-15

**Authors:** Fumihiko Urano, Bess A. Marshall, Stacy Hurst, Amy Robichaux-Viehoever, Saumel Ahmadi, Tamara Hershey, Gregory Van Stavern, Paulina Cruz Bravo, Jennifer Powers Carson, John Pesko, Kelly Fox, Nathalie Erpelding, Camille L. Bedrosian

**Affiliations:** 1 Department of Medicine,; 2 Department of Pathology & Immunology,; 3 Departments of Pediatrics and Cell Biology & Physiology,; 4 Department of Neurology,; 5 Departments of Psychiatry and Radiology, and; 6 Department of Ophthalmology & Visual Sciences, Washington University School of Medicine, St. Louis, Missouri, USA.; 7 Amylyx Pharmaceuticals, Inc., Cambridge, Massachusetts, USA.

**Keywords:** Endocrinology, Genetics, Cell stress, Clinical trials, Genetic diseases

## Abstract

PB&TURSO was associated with improved or stabilized pancreatic function, vision, and overall symptom burden in individuals with Wolfram syndrome, a rare and progressive degenerative disease.

**To the Editor:** Wolfram syndrome (WS) is a rare, progressive, monogenic disorder causing juvenile-onset, insulin-requiring diabetes; optic nerve atrophy; neurodegeneration; and premature death (1). Most cases involve biallelic, autosomal, recessive loss of function in *WFS1*, resulting in ER and mitochondrial dysfunction (2, 3). No approved treatments exist (1). An investigational oral combination of sodium phenylbutyrate and taurursodiol (PB&TURSO) targets dysfunctional ER and mitochondrial pathways to reduce pancreatic β cell and neuronal death (4, 5).

We report 24- and 48-week results from the ongoing HELIOS trial (ClinicalTrials.gov NCT05676034), a single-center, single-arm, open-label, phase II trial of PB&TURSO. Adults ≥ 17 years with genetically confirmed WS and insulin-requiring diabetes with residual β cell function were eligible. Further details are provided in Supplemental Methods.

Participant disposition and characteristics are summarized in Supplemental Figure 1 and Supplemental Table 1, respectively (supplemental material available online with this article; https://doi.org/10.1172/JCI198519DS1). All 12 enrolled participants received ≥1 dose and were included in safety and intent-to-treat (ITT) analyses. One participant was later determined to have confirmed pathogenic autosomal recessive *WFS1* mutation on only 1 allele and variant of uncertain significance on the other and did not exhibit typical WS phenotypic features; accordingly, the per-protocol population excluding this participant is considered the primary efficacy population.

The primary efficacy endpoint — C-peptide response at 120 minutes during a mixed-meal tolerance test (MMTT) — showed overall improvement from baseline at weeks 24 and 48. Among the endpoint components, C-peptide AUC increased in both ITT and per-protocol populations, and ΔC-peptide increased in the per-protocol population (Figure 1, A and B).

Secondary measures including HbA1c and time in target glucose range improved from baseline at weeks 24 and 48 (Figure 1, C and D). Best-corrected visual acuity trended toward stabilization over 48 weeks in both analysis sets (Figure 1E), consistent with periods of stability in natural history cohorts.

All participants with available data were classified as responders on both Participant and Clinician Global Impression of Change (PGI-C and CGI-C) scales at weeks 24 and 48 (Figure 1F). Qualitative interviews indicated meaningful improvements in diabetes and vision problems, the most bothersome pretrial symptoms (Supplemental Table 2), reinforcing clinical relevance of objective outcomes.

At the analysis cutoff (January 10, 2025), mean (range) treatment duration was 69.2 (34.6–91.4) weeks. Eleven participants experienced ≥1 treatment-emergent adverse events (TEAEs; Supplemental Table 3), all mild or moderate and mostly gastrointestinal. TEAEs led to treatment interruption or reduction in 3 participants each, but not to discontinuations. No serious TEAEs or deaths were reported.

Limitations include the open-label, single-arm design and small sample size, reflecting WS rarity. Nonetheless, objective improvements in C-peptide and HbA1c suggest that the stabilization or improvement observed was not due to design limitations. The trial focused on *WFS1* WS to reduce heterogeneity, though *CISD2* WS shares similar pathophysiology and may benefit. Marked genotype-phenotype variability (>200 *WFS1* variants) warrants cautious interpretation (6).

Overall, in the first 48 weeks of the open-label HELIOS trial, participants with WS treated with PB&TURSO demonstrated stabilization or improvement across multiple disease-related outcomes. Findings are notable given participant ages (all > 17 years) and the progressive nature of WS, making even slowed progression clinically meaningful (1). PB&TURSO was generally well tolerated, with mostly gastrointestinal adverse events and no serious TEAEs or discontinuations. Collectively, these results support continued development of PB&TURSO.

For detailed methods, information regarding sex as a biological variable, statistics, study approval, acknowledgments, author contributions, and Supporting Data Values, see the supplemental materials.

## Conflict of interest

FU has a sponsored research agreement and received material support from Prilenia Therapeutics and has received NIH grants, royalties from Novus Biologicals and Sana Biotechnology, licensing and/or consulting fees from Opris Biotechnologies and Emerald Biotherapeutics, and travel fees from Wolfram France, Wolfram UK, and Snow Foundation; serves unpaid advisory roles for Snow Foundation and Be A Tiger Foundation; holds US patents (9,891,231; 10,441,574; 10,695,324); and was President and shareholder of now dissolved CURE4WOLFRAM. BAM reports salary support from Amylyx Pharmaceuticals. SA reports salary support from an NIH KL2 award, research support from UpLifting Athletes, and travel support from the Epilepsy Foundation. TH reports salary support from Amylyx Pharmaceuticals, NIH grants, honoraria/travel fees from University of Southern California, and honoraria for grant reviews from Clayco Foundation. JPC reports salary support from NIH grant P30 DK020579, which supported some HELIOS testing. JP, KF, NE, and CLB are Amylyx employees and hold stock/options.

## Funding support

This work is the result of NIH funding, in whole or in part, and is subject to the NIH Public Access Policy. Through acceptance of this federal funding, the NIH has been given a right to make the work publicly available in PubMed Central.

- Amylyx Pharmaceuticals, Inc.
- NIH grant P30 DK020579.

## Supplementary Material

Supplemental data

Supporting data values

## Figures and Tables

**Figure 1:**
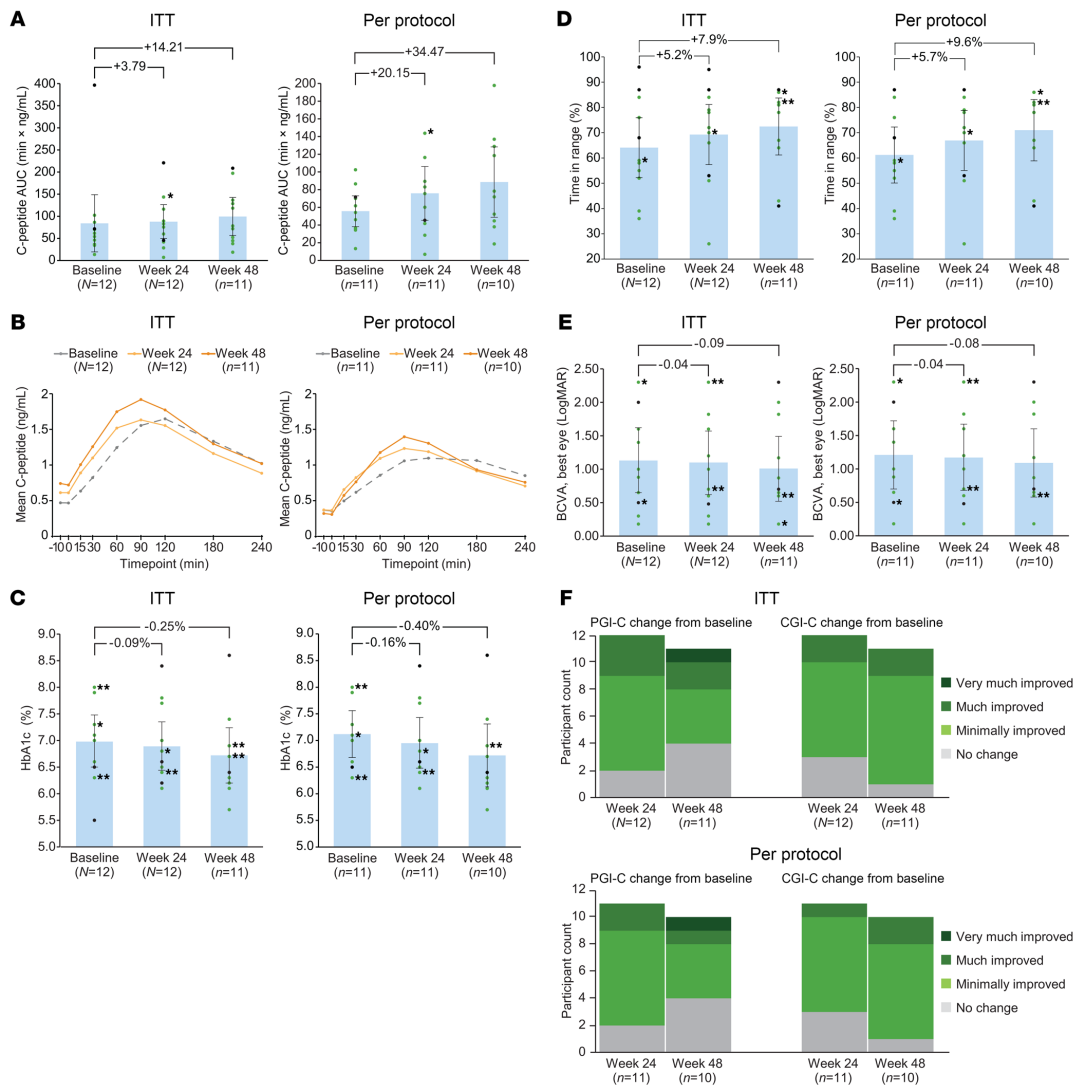
Clinical and functional outcomes. (**A**) Mean and participant-level trough-adjusted C-peptide AUC at 120 minutes into 240-minute MMTTs at baseline and weeks 24 and 48. All participants improved (green dots), except 1 per-protocol and 2 ITT participants (black dots). Asterisk denotes overlapping data points (both green). The *y* axis scales differ between graphs due to single ITT outlier. (**B**) Mean C-peptide levels during MMTTs at baseline and weeks 24 and 48 (95% CIs are provided in the Supporting Data Values file). (**C** and **D**) Summary and participant-level data for secondary glycemic control measures: HbA1c (**C**) and time in target glucose range (**D**; 70–180 mg/dL). (**E**) Best-corrected visual acuity (BCVA) as assessed by logarithm of the minimum angle of resolution (LogMAR) in the best eye. (**F**) Symptom burden at weeks 24 and 48 assessed via PGI-C and CGI-C. All measured participants were responders (defined by ratings of minimally, much, or very much improved or no change). Means (bars), 95% CIs (error bars), and mean changes (brackets) are shown in **A** and **C**–**E**. In participant-level data, green indicates improved or stable, and black indicates others. Single asterisks denote overlapping points of same color; double asterisks denote 1 green and 1 black.

## Version 1 05/15/2026

Electronic publication
