# Supplementary material for: Phase II trial of sodium phenylbutyrate and taurursodiol in Wolfram syndrome
Source: J Clin Invest. 2026 May 15;136(10):e198519. doi: 10.1172/JCI198519 (PMC13178640; doi:10.1172/JCI198519)
Supplement: Supplemental data [file jci-136-198519-s096.pdf]

Supplemental Appendix

Phase II trial of sodium phenylbutyrate and taurursodiol in Wolfram syndrome

Fumihiko Urano,[1,2] Bess A. Marshall,[3] Stacy Hurst,[1] Amy Robichaux-Viehoever,[4] Saumel Ahmadi,[4] Tamara Hershey,[5] Gregory Van Stavern,[6] Paulina Cruz Bravo,[1] Jennifer Powers Carson,[1] John Pesko,[7] Kelly Fox,[7] Nathalie Erpelding,[7] Camille L. Bedrosian[7]

[1]Department of Medicine, [2]Department of Pathology & Immunology, [3]Departments of Pediatrics and Cell Biology & Physiology, [4]Department of Neurology, [5]Departments of Psychiatry and Radiology, and [6]Department of Ophthalmology & Visual Sciences, Washington University School of Medicine, St. Louis, Missouri, USA; [7]Amylyx Pharmaceuticals, Inc., Cambridge, Massachusetts, USA

Table of contents

1	Acknowledgements ……………………………………………………………………..	3
2	Author contributions …………………………………………………………………..	3
3	Supplemental methods	
	Section 3.1. Sex as a biological variable ……………………………………..……….....	4
	Section 3.2. Trial design ……………………………………………………………….…..	4
	Section 3.3. Participants …………………………………………………………..….	4
	Section 3.4. Trial eligibility criteria ………………………………………………….	5
	Section 3.5. Interventions and procedures …………………………….………..	10
	Section 3.6. Trial endpoints and assessments ………………………………………..	11
	Section 3.7. Statistics ……………………………………………………………………...	14
	Section 3.8. Study approval ……………………………………………………….	15
	Section 3.9. Data availability ……………………………………..…………….	15
4	Supplemental tables	
	Table 1. Demographic and clinical characteristics ………………………….…………..…	17
	Table 2. Positive changes in Wolfram syndrome-related symptoms and complications according to qualitative participant interviews ………………………………………	19
	Table 3. Summary of treatment-emergent adverse events ………………………………	21
5	Supplemental figure	
	Figure 1. Trial participant flow ………………………….…………………………...	23
6	References	24

1. Acknowledgments

The authors extend their deepest gratitude to the people living with Wolfram syndrome who participated in the HELIOS trial, as well as their loved ones, and the entire Wolfram syndrome community for their support of this trial. The authors also thank the Wolfram Syndrome Clinic and research team at Washington University School of Medicine, especially those who contributed to this trial, including Alexis McKee, MD, Yunshuo Tang, MD, PhD, Leanne Stunkel, MD, Cris Brown, Gabriel Skinner, Mary Jane Clifton, Kathryn Bohnert, Anna Asako, Elizabeth Sellitto, Joshua Chen, Caroline Raso, Abby Tang, Rachel Reiss, Sarah Ning, and Shrinivas Bimal. The sponsor, Amylyx Pharmaceuticals, Inc., was involved in the trial design, data analysis, and manuscript development and provided the active drug. C-peptide and glycated hemoglobin testing was performed in the Washington University Diabetes Research Center's Translational Diagnostics Core and subsidized by the National Institutes of Health grant P30 DK020579. Medical writing support was provided by Lara Primak, MD, and Nikhilesh Sanyal, PhD, of Precision AQ and funded by the sponsor. All authors constructively reviewed the manuscript and provided approval for submission.

2. Author contributions

FU participated in the conceptualization and design of the trial and the analysis of trial data, contributed to data acquisition and trial oversight, and acquired trial funding, which was provided by Amylyx Pharmaceuticals, Inc. BAM provided clinical oversight and endocrinology assessments. SH contributed to data acquisition and trial oversight. AR-V and SA conducted neurological assessments. TH conducted neuropsychological assessments. GVS conducted ophthalmological assessments. PCB provided endocrinology assessments. JPC provided clinical

laboratory oversight. JP participated in the conceptualization and design of the trial and the analysis of trial data. KF participated in the conceptualization and design of the trial and the analysis of trial data and drafted the manuscript. NE and CLB participated in the conceptualization and design of the trial and the analysis of trial data. All authors critically reviewed the manuscript and approved the final submitted version.

3. Supplemental methods

3.1. Sex as a biological variable

The trial enrolled male and female participants. Sex was not considered as a biological variable.

3.2. Trial design

HELIOS (NCT05676034) is an ongoing, single-arm, open-label trial performed at a single site in the United States (Washington University, St. Louis, Missouri) that commenced in March 2023. HELIOS was originally designed as a 24-week study. However, the study duration was extended to allow for supplementary evaluation of longitudinal efficacy and safety. The revised overall study duration is up to 212 weeks, consisting of a screening period of up to 4 weeks, a 48-week treatment period, a 160-week extension treatment period, and a safety follow-up visit occurring 4 weeks after the end-of-treatment visit. The results presented are from an analysis at a cutoff date of January 10, 2025 (minimum and maximum treatment duration of 34.6 weeks and 91.4 weeks, respectively).

3.3. Participants

Adults ≥ 17 years of age with a definitive diagnosis of Wolfram syndrome were eligible for the trial. Definitive diagnosis was determined by documentation of a functionally relevant,

autosomal recessive variant affecting both alleles of the *WFS1* gene, either on historical testing or from a qualified laboratory at screening. Participants were also required to have insulin-requiring diabetes mellitus (DM) due to Wolfram syndrome at baseline, but with residual β cell function as defined by a stimulated C-peptide level ≥ 0.2 ng/mL at screening, to ensure a valid response to mixed-meal tolerance test (MMTT). For all allowable concomitant medications, participants were to remain on stable regimens for ≥ 30 days prior to their screening visit; in addition, all allowable concomitant medications were to remain at stable doses for the duration of the trial unless a change was medically necessary. Glucagon-like peptide-1 receptor agonist use was not permitted during the first 48 weeks of the trial.

Full inclusion and exclusion criteria are summarized below.

3.4. Trial eligibility criteria

Inclusion criteria

To be eligible for entry into the study, participants must have met all of the following criteria:

1. Provided a signed informed consent form (ICF) and had the mental capability to understand the ICF. If participant was unable to sign the ICF, the ICF must have been signed by a representative in accordance with local regulatory requirements
2. Definitive diagnosis of Wolfram syndrome, as determined by a documented functionally relevant recessive mutation on both alleles of the *WFS1* gene based on historical test results (if available) or from a qualified laboratory at screening
3. Stimulated C-peptide level of ≥ 0.2 ng/mL during the screening visit
4. Insulin-dependent DM due to Wolfram syndrome

5. Aged ≥ 17 years at the time of providing written informed consent
6. Willing to wear a continuous glucose monitoring (CGM) device through week 208/end of trial or until the safety follow-up visit, as applicable to the participant
7. For female participants:
    a. If of childbearing potential (i.e., not postmenopausal for ≥ 1 year and not surgically sterile), must have agreed to use adequate birth control* for the duration of the study and for 6 months after the last dose of study drug
    b. Must not have been planning to become pregnant for the duration of the study and for 6 months after the last dose of study drug
8. For male participants:
    a. Must have agreed to practice contraception* for the duration of the study and for ≥ 6 months after the last dose of study drug
    b. Must not have been planning to father a child or provide sperm for donation for the duration of the study and for 6 months after the last dose of study drug

*Acceptable birth control methods for use in the study include hormonal methods (e.g., birth control pills, patches, injections, vaginal ring, implants), barrier methods (e.g., condom, diaphragm) used with a spermicide, intrauterine device, abstinence (i.e., no heterosexual sexual intercourse), and sexual intercourse with only a unique partner who is surgically sterile (male) or not of childbearing potential (female).

<u>Exclusion criteria</u>

Participants were not eligible for the study if they met any of the following criteria during screening evaluations or as otherwise noted:

1. Clinically significant non–Wolfram syndrome–related central nervous system involvement that was judged by the Investigator to likely interfere with the accurate administration and interpretation of protocol assessments
2. Clinically significant, unstable medical condition (other than Wolfram syndrome) that would pose a risk to the participant if they were to participate in the study, according to the Investigator's judgment
3. Clinically significant (in the opinion of the Investigator) infection or inflammation at the time of screening or admission. (However, if the infection or inflammation had been cured, the participant could be rescreened.)
4. Acute gastrointestinal symptoms (e.g., nausea, vomiting, diarrhea) at the time of screening or admission
5. Presence of pathologies that could alter the enterohepatic circulation of bile acids (e.g., ileal resection and stoma, regional ileitis)
6. Presence of unstable psychiatric disease, cognitive impairment, dementia, or substance abuse that would impair the ability of the participant to provide informed consent and follow instructions, according to the Investigator's judgment
7. Any major surgery within 4 weeks of screening
8. Inability to comply with the protocol (e.g., due to a clinically relevant medical condition making implementation of the protocol difficult, an unstable social situation, known clinically significant psychiatric or behavioral instability, inability to travel to the trial site as required for study evaluations, or unlikeliness to complete the study for other reasons), as determined by the Investigator
9. History of known allergy to sodium phenylbutyrate (PB) or bile salts

10. Abnormal liver function, defined as aspartate transaminase and/or alanine transaminase more than 3 times the upper limit of the normal
11. Renal insufficiency, as defined by estimated glomerular filtration rate < 60 mL/min/1.73 m^2
12. Ongoing anemia with hemoglobin concentration < 10.0 g/dL
13. Pregnant or currently breastfeeding at screening
14. Biliary disease that may lead to biliary obstruction or impede biliary flow, including active cholecystitis, primary biliary cirrhosis, sclerosing cholangitis, gallbladder cancer, gallbladder polyps, gangrene of the gallbladder, or abscess of the gallbladder
15. Any history of heart failure per New York Heart Association criteria
16. Personal history of breast and/or ovarian cancer or family (i.e., biological parents, siblings, or avunculi) with a history or current diagnosis of either hormonal or postmenopausal breast or ovarian cancer
17. Under a severe salt restriction, in which the added salt intake due to study treatment would put the participant at risk in the Investigator's judgment
18. Treatment with any investigational drug or device within the 30 days (or 5 half-lives, whichever is longer) prior to the first dose of study drug on day 1
19. Blood product transfusion within 90 days prior to screening
20. Prior treatment with gene or cellular therapy
21. Evidence of organ dysfunction or any clinically significant deviation from normal in physical examination, vital signs, or clinical laboratory determinations beyond what is consistent with the target population in the opinion of the Investigator

22. Clinically significant abnormality on 12-lead electrocardiogram (ECG) prior to study drug administration, confirmed on repeat ECG
23. Any history of clinically significant suicidal ideation or behavior within 1 year of screening, as determined by the Investigator
24. Anything that, in the opinion of the Investigator, precluded the participant's full compliance with or completion of the study
25. Current treatment with, previous treatment (within the 30 days prior to screening) with, or planned exposure to any of the following prohibited medications:
    a. Reldesemtiv
    b. Inosine
    c. Acetyl-L-carnitine
    d. Methylcobalamin (if administered at doses $\geq$ 25 mg/week)
    e. Antisense therapy
    f. Histone deacetylase inhibitors, including valproate, vorinostat, romidepsin, chidamide, panobinostat, lithium, butyrate, and suramin
    g. Fludrocortisone
    h. Probenecid for potential kidney interaction
    i. Mexiletine (except if used for management of cramps and fasciculation)
    j. Antacids containing aluminum hydroxide or smectite (aluminum oxide) within 2 hours before or after administration of trial drug, as they may inhibit absorption of taurursodiol (TURSO)
    k. Bile acid sequestrants, including products containing cholestyramine, colesevelam, and colestipol

l. Bile acid and derivatives, whether as a supplement or an over-the-counter or prescription drug, including ursodeoxycholic acid, TURSO, and tauroursodeoxycholic acid
m. Neuroleptics, including chlorpromazine, fluphenazine, loxapine, perphenazine, thioridazine, thiothixene, trifluoperazine, and haloperidol
n. Benzodiazepines/gamma-aminobutyric acid (GABA) agonists, including chlordiazepoxide, diazepam, midazolam, flurazepam, temazepam, meprobamate, and triazolam
o. Substrates of CYP2C8, CYP2C9, CYP2C19, CYP2B6, CYP1A2, and CYP3A4/5, including rasagiline, masitinib, pioglitazone, olanzapine, carbamazepine, methadone, warfarin, quinidine, and phenytoin
p. Substrates of P-glycoprotein and breast cancer resistance protein (BCRP), including digoxin, apixaban, dabigatran, and tamoxifen
q. Substrates of organic anion transporter (OAT) 1 and OAT3, including penicillins and methotrexate
r. Inhibitors of OATP1B3, including cyclosporin, gemfibrozil, and rifampicin

3.5. Interventions and procedures

PB and TURSO was provided as a powder (3 g PB and 1 g TURSO) in single-use sachets. Details regarding the administration of PB and TURSO are published elsewhere (1). Participants were instructed to take 1 sachet of trial drug per day in the morning through day 21 and then increase to 2 sachets per day (1 in the morning and 1 in the evening) thereafter, if no intolerable side effects occurred. During the treatment period of the trial, clinic visits were conducted at

baseline and weeks 12, 24, 36, and 48. Additional remote (phone/telehealth) visits occurred at weeks 1 and 2 and then every 4 weeks starting at week 4.

Investigators were instructed that they could initially manage treatment-emergent adverse events (TEAEs) that were intolerable to the participant and possibly related to trial drug in their opinion (including, but not limited to, persistent diarrhea, vomiting, nausea, signs of dehydration, and increase in serum creatinine or liver enzymes) with stepwise reduction(s) in trial drug dose. The first-level dose adjustment was reduction to 1 sachet of trial drug per day. If this first-level dose reduction did not result in improvement within 7 to 14 days, the dose could be further reduced to 1 sachet of trial drug once every 2 days. The Investigator could decide at any time to interrupt treatment.

If a participant demonstrated treatment-emergent signs of neurotoxicity that were possibly related to trial drug in the opinion of the Investigator, the Investigator could consider a dose reduction or interruption.

3.6. Trial endpoints and assessments

Efficacy results through the 48-week treatment period are reported. The primary efficacy endpoints assessed the effect of PB and TURSO on residual pancreatic β cell function as measured by C-peptide levels in response to MMTTs (2). The night before the MMTT, participants were instructed to implement appropriate exogenous insulin dosing modifications per Investigator instruction and fasted from midnight until the test the following morning. Participants were instructed not to administer short-acting insulin, metformin, or sodium-glucose

cotransporter 2 inhibitors before the MMTT. The mixed meal consisted of 6 mL/kg (maximum 360 mL) of Boost Original (Société des Produits Nestlé S.A., Vevey, Switzerland), consumed within a 5-minute period. PB and TURSO was administered prior to the test. Blood samples for C-peptide and glucose levels were drawn at minutes -10, 0, 15, 30, 60, 90, 120, 180, and 240 ±5 minutes, with the 0-minute collection time coinciding with the start of the participant's ingestion of the mixed meal. C-peptide levels were determined by electrochemiluminescence immunoassay (Elecsys® C-Peptide; Roche Diagnostics) (3).

C-peptide response was assessed throughout the MMTT using 2 parameters: i) the C-peptide AUC, using the trapezoidal method and calculated after trough adjustment (i.e., subtracting the value at time 0 minutes so each curve was centered at value 0 at time 0 minutes), and ii) the ΔC-peptide, defined as the absolute change in C-peptide level from time 0. The primary efficacy endpoints evaluated the change from baseline to week 24 in both parameters, using the 0- to 120-minute interval of the MMTT as the primary metric of interest based on the typical timing of C-peptide response in people with monogenic DM (4). The C-peptide responses during other intervals of the MMTT were also investigated as nonprimary endpoints.

Secondary efficacy endpoints assessed included measures of glycemic control and best-corrected visual acuity (BCVA). Specifically, HbA1c change from baseline and time in target range (70-180 mg/dL), as measured by CGM, were the key measures of glycemic control evaluated. BCVA change from baseline was measured on the logarithm of the minimum angle of resolution (LogMAR) scale using the Snellen chart; values were taken for each eye after correction, with a possible range of 0 LogMAR units, representing perfect vision, to +3 LogMAR units,

representing no light perception (5, 6). All vision-related assessments were performed by a neuro-ophthalmologist (GVS) and/or clinic staff.

Exploratory endpoints assessed during the treatment period included the change from baseline in additional MMTT C-peptide measures and in the Participant Global Impression of Change (PGI-C) and Clinician Global Impression of Change (CGI-C) scales. The PGI-C and CGI-C use a 7-level Likert-scale ordinal response to assess improvement or deterioration in symptoms relative to an anchor point (in this case, since initiating study drug) (7). Scores range from 1 (very much improved) to 7 (very much worse). Scores of 1 (very much improved) through 4 (no change) were used to define responders in this trial, given the progressive nature of Wolfram syndrome. The change in participant experience of Wolfram syndrome was also assessed via qualitative on-study interviews. Participants who reached at least 24 weeks of treatment could participate in a 60-minute interview using a semistructured interview guide developed specifically for the trial by a third-party vendor. These interviews were conducted by trained moderators. Interviews were audio recorded and transcribed for analysis. Using a thematic qualitative data analysis approach, interview transcripts were coded to identify key concepts (e.g., symptoms and impacts) and dominant trends in the interview data. Data were analyzed and summarized (e.g., in text as well as in frequencies and percentages) in aggregate for the overall sample.

Safety assessments included the incidence and severity of TEAEs and serious TEAEs, the incidence of abnormalities in clinical laboratory assessments, and withdrawal from the trial due to TEAEs through the analysis cut-off date. Any events that may have been attributable to

progression or defining manifestations of Wolfram syndrome were reviewed by the Investigator and site staff to determine whether they met criteria for reporting as a TEAE.

3.7. Statistics

A total sample size of up to 12 participants was planned. The sample size was estimated based on enrollment feasibility assuming a 15% dropout rate and the level of precision the study would provide around the estimated mean change from baseline in ΔC-peptide at week 24 (ΔΔC-peptide). Assuming observation of a mean ΔΔC-peptide of 0.12 ng/mL and a standard deviation of 0.18 ng/mL, the corresponding 95% CI was determined to be 0.006 to 0.234. Hence, a sample size of 12 participants was determined to provide enough precision such that the anticipated lower confidence limit was > 0 (where ΔΔC-peptide=0 ng/mL suggests no treatment effect and ΔΔC-peptide > 0 ng/mL suggests an improvement).

The primary population for efficacy analyses was the per-protocol population, comprising all participants with a definite diagnosis of Wolfram syndrome as defined by documented biallelic pathogenic mutations in the *WFS1* gene on genetic review, per the trial inclusion criteria. Intent-to-treat efficacy and safety analyses incorporated all participants who received ≥ 1 dose of study drug.

No formal inferential procedures were pre-specified for this trial; all statistical summaries are intended to be purely descriptive in nature. For C-peptide, absolute value and change from baseline were summarized by visit. The primary endpoint measures of C-peptide response were reported as mean change from baseline with the associated 95% CIs. For the secondary endpoint

measures of HbA1c, time in target range based on CGM, and visual acuity, absolute value and change from baseline were summarized by visit and reported as mean change from baseline with the associated 95% CI. In the case of visual acuity, the mean change from baseline is reported for the best eye, as represented by the lowest (i.e., best) LogMAR score for a participant at each assessment. Changes in the exploratory endpoint measures of PGI-C and CGI-C score were summarized by the number and percentage of participants responding in each category of response at each assessment. Extent of exposure to PB and TURSO was assessed via the number of participants who received ≥ 1 dose and the duration of exposure. TEAEs were tabulated using descriptive summary statistics.

3.8. Study approval

The trial is being conducted in compliance with the Good Clinical Practice guideline of the International Council for Harmonisation of Technical Requirements for Pharmaceuticals for Human Use, the ethical principles of the Declaration of Helsinki, and applicable local regulations. The trial protocol was approved by the Institutional Review Board at the investigational site (Washington University IRB ID: 202409020). Written informed consent was obtained from each participant.

3.9. Data availability

Values for all data represented in graphs and as mean (95%CI) are reported in the Supporting Data Values file. Restrictions apply to the availability of some or all data that were generated or analyzed during this study to preserve participant confidentiality or because they were used

under license. Upon request, the corresponding author will detail the restrictions and any conditions under which access to some data may be provided.

4. Supplemental tables

Table 1. Demographic and clinical characteristics

	**Intent-to-treat participants (*N*=12)**	**Per-protocol participants (*n*=11)**
Age (yr)		
Mean (SD)	26.6 (8.3)	25.9 (8.3)
Median (range)	24.5 (18–39)	24.0 (18–39)
Sex (no. [%])		
Female	10 (83.3)	9 (81.8)
Male	2 (16.7)	2 (18.2)
Race (no. [%])		
White	12 (100)	11 (100)
Age of Wolfram syndrome diagnosis (yr)		
Mean (SD)	21.0 (10.4)	19.9 (10.2)
Median (range)	20.5 (8.0–36.0)	16.0 (8.0–36.0)
Time since Wolfram syndrome diagnosis (yr)		
Mean (SD)	6.0 (5.2)	6.4 (5.3)
Median (range)	5.1 (0.4–14.5)	7.4 (0.4–14.5)
Age of onset (yr)[A]		
Diabetes mellitus		
n	12	11
Mean (SD)	10.9 (7.9)	8.9 (4.0)
Median (range)	9.0 (2.8-33.0)	9.0 (2.8-17.0)

Arginine vasopressin deficiency		
n	4	4
Mean (SD)	13.5 (7.3)	13.5 (7.3)
Median (range)	11.3 (7.5–24.0)	11.3 (7.5–24.0)
Vision loss		
n	12	11
Mean (SD)	13.8 (8.1)	12.4 (6.9)
Median (range)	11.5 (5.2–29.0)	11.0 (5.2–28.0)
Hearing loss		
n	5	4
Mean (SD)	18.4 (11.1)	14.5 (7.9)
Median (range)	16.0 (7.3–34.0)	12.8 (7.3–25.0)
Concomitant diabetes medications, *n* (%)		
Insulins and analogues	12 (100)	11 (100)
Insulin lispro	8 (66.7)	8 (72.7)
Insulin glargine	5 (41.7)	4 (36.4)
Insulin aspart	2 (16.7)	2 (18.2)
Insulin degludec	1 (8.3)	1 (9.1)
Insulin lispro-aabc	1 (8.3)	1 (9.1)
Metformin	3 (25.0)	2 (18.2)

[A]Among participants reporting the respective disease manifestations, as indicated by the *n* values.

Table 2. Positive changes in Wolfram syndrome–related symptoms and complications according to qualitative participant interviews

Symptom/complication[A]	Number of participants reporting before trial[B]	Number of participants reporting positive change (no. [%])[C]	Number of participants reporting change was important (no. [%])[D]
Vision problems	11	7 (63.6)	7 (100)
Bladder issues (e.g., pain, incontinence)	11	5 (45.5)	4 (80.0)
Insulin-requiring DM	11	8 (72.7)	8 (100)
Fatigue	6	4 (66.7)	4 (100)
Problems swallowing (e.g., choking episodes)	5	3 (60.0)	3 (100)
Headache/migraine[E]	4	3 (75.0)[E]	3 (100)
Speech problems	3	2 (66.7)	2 (100)
Tics	3	2 (66.7)	2 (100)
Ataxia	3	1 (33.3)	1 (100)
Problems with mobility/walking	2	2 (100)	2 (100)
Loss of smell	2	2 (100)	2 (100)
Numbness	2	2 (100)	1 (50.0)
Heat intolerance	2	1 (50.0)	1 (100)

Dystonia	2	1 (50.0)	1 (100)
Difficulty thinking or concentrating	1	1 (100)	1 (100)
Extreme thirst and substantial fluid intake	1	1 (100)	1 (100)
Acute pancreatitis	1	1 (100)	1 (100)

[A]In descending order of frequency before trial start. [B]Of a total of 11 participants who completed the qualitative interview. [C]Among those participants reporting the symptom/complication before the trial. [D]Among those participants reporting the symptom/complication before the trial who reported a positive change in the symptom/complication on treatment. [E]Includes 1 participant who reported ear pain in conjunction with headaches who reported improvements in both symptoms.

Table 3. Summary of treatment-emergent adverse events[A]

**Variable**	**PB and TURSO (*N*=12)[B]**
≥ 1 TEAEs (no. [%])	11 (92)
Grade 1 (mild)	11 (92)
Grade 2 (moderate)	4 (33)
Grade 3 (severe)	0 (0)
Grade 4 (life-threatening)	0 (0)
Grade 5 (fatal)	0 (0)
Relationship to PB and TURSO[C]	
Not related	3 (25)
Unlikely related	10 (83)
Possibly related	10 (83)
Probably related	1 (8)
Definitely related	0 (0)
PB and TURSO interrupted due to TEAE (no. [%])	3 (25)
PB and TURSO dose reduced due to TEAE (no. [%])	3 (25)
PB and TURSO discontinued due to TEAE (no. [%])	0 (0)
≥ 1 serious TEAE (no. [%])	0 (0)
TEAEs occurring in > 10% of participants (no. [%])[D]	
Diarrhea	7 (58)
Vomiting	4 (33)
Arthralgia	3 (25)
Sinusitis	3 (25)

Abdominal pain upper	2 (17)
Constipation	2 (17)
Nausea	2 (17)
Ear infection	2 (17)
Headache	2 (17)
Hypoesthesia	2 (17)
Depression	2 (17)
Mood swings	2 (17)

[A]Through January 10, 2025 (analysis cut-off date). [B]Data represent the number and percentage of participants in the safety population experiencing each of the indicated events. The safety population included all participants who received ≥ 1 dose of PB and TURSO. [C]The relatedness of TEAEs to PB and TURSO was determined by the site Investigator. [D]TEAEs were classified according to system organ class and preferred term in the *Medical Dictionary for Regulatory Activities*, version 25.1 (or later).

5. Supplemental figure

Figure 1. Trial participant flow

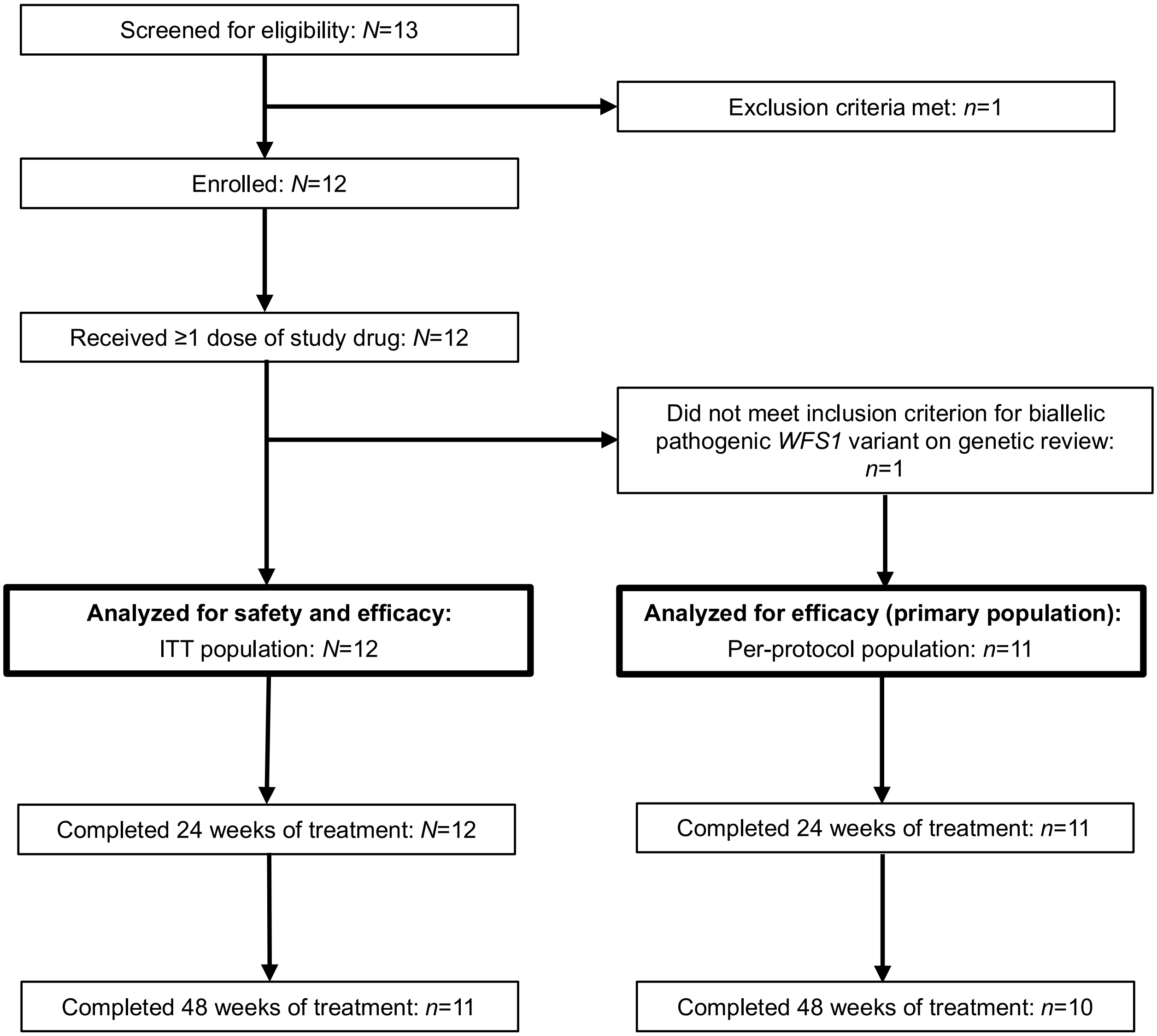

6. References

1. Paganoni S, et al. Trial of sodium phenylbutyrate–taurursodiol for amyotrophic lateral sclerosis. *N Engl J Med.* 2020;383(10):919–930.

2. Buss RW, et al. Mixed meal tolerance test and reactive hypoglycemia. *Horm Metab Res.* 1982;14(6):281–283.

3. Roche Diagnostics. Elecsys® C-Peptide. https://elabdoc-prod.roche.com/eLD/web/global/en/products/CPS_000460. Accessed August 11, 2025.

4. Stankute I, et al. Kinetics of C-peptide during mixed meal test and its value for treatment optimization in monogenic diabetes patients. *Diabetes Res Clin Pract.* 2021;178:108938.

5. De Silva SR, et al. Visual acuity by decade in 139 males with *RPGR*-associated retinitis pigmentosa. *Ophthalmol Sci.* 2023;4(2):100375.

6. Holladay JT. Visual acuity measurements. *J Cataract Refract Surg.* 2004;30(2):287–290.

7. Swanenburg J, et al. Patients' and therapists' perception of change following physiotherapy in an orthopedic hospital's outpatient clinic. *Physiother Theory Pract.* 2015;31(4):293–298.
